# Surface Modification of a Nanoporous Carbon Photoanode upon Irradiation

**DOI:** 10.3390/molecules21111611

**Published:** 2016-11-23

**Authors:** Alicia Gomis-Berenguer, Inmaculada Velo-Gala, Enrique Rodríguez-Castellón, Conchi O. Ania

**Affiliations:** 1 ADPOR Group, Instituto Nacional del Carbón (INCAR, CSIC), Oviedo 33001, Spain; i.velo@incar.csic.es; 2 Department Química Inorgánica, Facultad de Ciencias, Universidad de Málaga, Málaga 29071, Spain; castellon@uma.es

**Keywords:** nanoporous carbons, simulated solar light, photoanodes, surface modification

## Abstract

The photocorrosion of a nanoporous carbon photoanode, with low surface functionalization and high performance towards the photoelectrochemical oxidation of water using simulated solar light, was investigated. Two different light configurations were used to isolate the effect of the irradiation wavelength (UV and visible light) on the textural and chemical features of the carbon photoanode, and its long-term photocatalytic performance for the oxygen evolution reaction. A complete characterization of the carbon showed that the photocorrosion of carbon anodes of low functionalization follows a different pathway than highly functionalized carbons. The carbon matrix gets slightly oxidized, with the formation of carboxylic and carbonyl-like moieties in the surface of the carbon anode after light exposure. The oxidation of the carbon occurred due to the photogeneration of oxygen reactive species upon the decomposition of water during the irradiation of the photoanodes. Furthermore, the photoinduced surface reactions depend on the nature of the carbon anode and its ability to photogenerate reactive species in solution, rather than on the wavelength of the irradiation source. This surface modification is responsible for the decreased efficiency of the carbon photoanode throughout long illumination periods, due to the effect of the oxidation of the carbon matrix on the charge transfer. In this work, we have corroborated that, in the case of a low functionalization carbon material, the photocorrosion also occurs although it proceeds through a different pathway. The carbon anode gets gradually slightly oxidized due to the photogeneration of O-reactive species, being the incorporation of the O-groups responsible for the decreased performance of the anode upon long-term irradiation due to the effect of the oxidation of the carbon matrix on the electron transfer.

## 1. Introduction

Solar energy conversion and storage is a research topic at the forefront of technology that has attracted the attention of scientists across various disciplines. The use of sunlight for the catalytic splitting of water to produce hydrogen and oxygen is a key process for solar fuel production systems towards a sustainable utilization of electricity generated from renewable energy resources. From the large-scale application point of view, water breakdown by electro- and/or photo-assisted methods is hampered by the low overall efficiency of the reaction and the scarcity and high cost of the precious metals typically used as catalysts and/or photocatalysts [1,2,3,4,5].

Most commonly used materials in photocatalytic processes are semiconductors, however they usually present low photonic efficiencies under solar light. For instance, one of the benchmark materials, TiO_2_, does not to absorb photons with wavelengths longer than 387 nm (i.e., 3.2 eV), thus much of the sunlight is wasted as the exploitation is reduced to ca. 4%. For these reasons, research on the use of abundant materials with improved harvesting features across the visible region that can serve as efficient catalysts in such systems is currently receiving much attention [6,7,8]. Extensive work has been carried out on the use of carbon materials for this purpose, mainly coupling different forms of carbons with TiO_2_ and other photocatalysts [9,10,11]. Among all types of carbons, graphene has been intensively investigated as a photocatalyst and it has been known for some time that either by itself or combined to other photoactive materials, graphene is able to catalyze the water splitting reaction (mainly oxygen reduction reactions) in alkaline media [9,12,13,14,15]. Much research is also being done on exploring the photocatalytic activity of some other metal-free carbon photoanodes [16,17,18,19].

On the other hand, we have recently reported the photochemical activity of metal-free nanoporous carbons in the absence of semiconductors under various irradiation conditions [20,21,22,23,24]. Despite the similarities between the structural and chemical features of graphene and those of nanoporous carbons, the photochemical activity of the latter has not received much attention.

Following the application of nanoporous carbons in photochemical reactions, we have recently explored the use of nanoporous carbons as photoanodes in photoelectrochemical water splitting [23,25,26]. Data showed that surface functionalization is an important factor for the performance of the carbon anode, with carbons showing a rich surface chemistry among the most performing materials. However, the characterization of the photoanodes after illumination showed the photo- and electro-corrosion of the anodes, attributed to redox reactions of O-, N-, and S-surface groups of the carbon matrix [23,26] giving rise to the consumption of the active sites and charge transfer limitations.

To further clarify the issue of photocorrosion observed in carbon anodes, we have investigated a nanoporous carbon with low functionalization and investigated its ability to photocatalysis the oxidation of water after long irradiation periods, as well as the extent of surface photocorrosion due to the long-term light exposure. An extensive surface and textural characterization of the carbon anodes after illumination under different conditions was made, trying to link the effect of the irradiation wavelength with the deactivation of the anode under long illumination periods, and particularly with the photoinduced modifications in its specific textural, chemical, and structural features.

## 2. Results and Discussion

In a previous study, we reported the performance of nanoporous carbon photoanodes for the photocatalytic and photoelectrochemical oxidation of water under solar light, using carbons from various origins and rich surface chemistry [23,26]. Data showed that the performance of the carbon anodes was strongly influenced by the surface functionalization of the carbon materials, both in terms of the reaction yield (i.e., photocatalytic water splitting yield) and the stability of the electrodes after long illumination exposures, with a marked deactivation in the case of carbons showing a complex surface chemistry (highly functionalized carbons with N-, O-, and S-containing groups ranging from 10–12 at. % oxygen, 1–2 at. % nitrogen and 0.2–0.32 at. % sulfur) due to photoinduced reduction of surface groups of the anodes.

In this work, we extend this study to nanoporous carbons with low surface functionalization, where the deactivation (if any) should not be attributed to the redox reactions involving the heteroatoms and surface groups decorating the carbon matrix. Details on the textural and chemical characteristics of the nanoporous carbons used as photoanodes, as well as their performance towards the photocatalytic and photoelectrochemical oxidation of water using simulated solar light have been presented in detail elsewhere [20,23,26]. We herein reintroduce some details on their textural and chemical properties for better comprehension of the response and surface modification of the carbon photoanodes when exposed to solar irradiation.

### 2.1. Photocatalytic Performance of the Nanoporous Carbon Anodes

**Figure 1:**
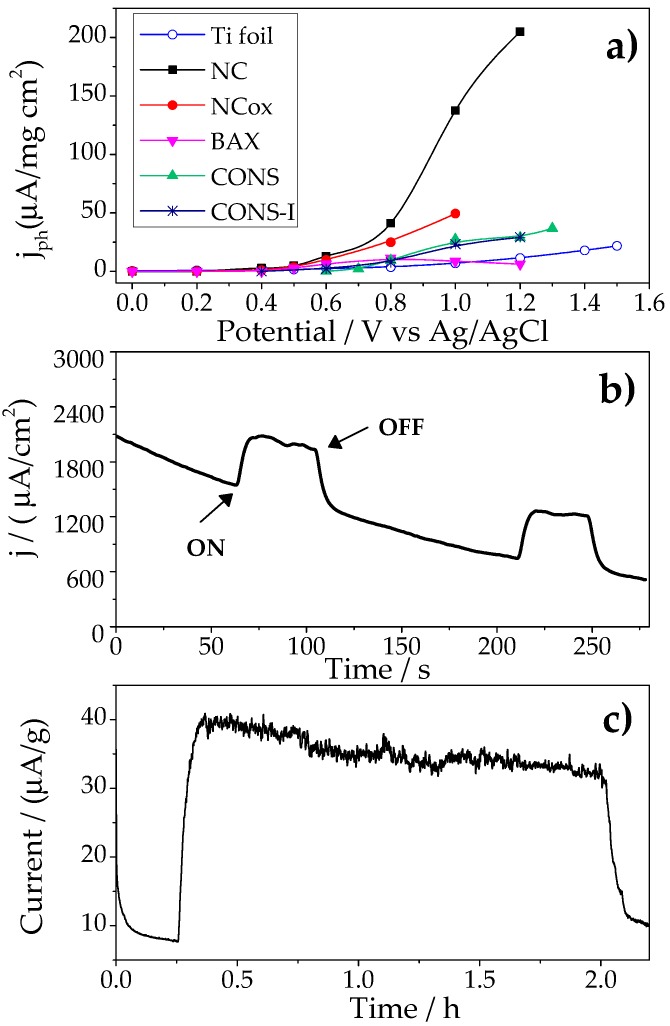
(**a**) Photocurrent densities of different samples (NC, NCox, BAX, CONS, and CONS-I) at various potentials (0.0–1.5 V vs. Ag/AgCl), data corresponding to the bare titanium foil current collector is also included for comparison; (**b**) chronoamperometric response; and (**c**) long-term performance of NC photoanode upon on/off illumination of simulated solar light irradiation at 1 V vs. Ag/AgCl.

Figure 1a shows the comparison of the photocurrent generation at different bias potential (between 0 and +1.5 V vs. Ag/AgCl) on various carbon photoanodes with different chemical composition and porosity [23,26]. A complete characterization of the carbons has been reported and discussed in those previous works, herein we reintroduce some date for comprehension purposes (Table 1 and Figures S1–S4 in the Supplementary Materials). Briefly, with the exception of sample NC, the investigated carbons displayed high functionalization with O-, N-, and S-containing groups. Their porous features were also very different; as seen in Table 1, CONS can be considered as a nonporous carbon whereas CONS-I is a micro/mesoporous material with a marked level of microporosity. In contrast, BAX, NC, and NCox displayed well-developed porosity, with a predominance of mesopores in the case of the wood-based carbon (BAX).

For all the carbons, the transient photocurrent curves upon on/off illumination presented a square-shaped profile (Figure 1b), with a prompt initial rise followed by smooth fall until a steady-state regime was achieved; the anodic photocurrent retracted to original values once the illumination was turned off. The photocurrent response was stable and reproducible during several on/off short cycles (ca. 120 s of illumination). Since water molecules are the only scavengers in the reaction medium, the anodic photocurrent corresponds to the oxidation of water upon irradiation of the carbon photoanodes; this was also corroborated by measuring the O_2_ concentration in the electrolyte using a sensor.

The potential onset of the photocurrent was quite similar for all the carbon anodes varying between +0.6 and +0.8 V vs. Ag/AgCl (Figure 1a); these values were clearly lower than that of the bare current collector. Current densities between 100–200 μA/mg·cm^2^ were obtained for the highest potentials and the best performing photoanode. Interestingly, a higher photocurrent density was obtained for the nanoporous carbon with a low surface functionalization (sample NC). This contrasts with our previous study [26] where the photochemical response of carbon photoanodes, showing a complex surface chemistry, was linked to the presence of N-, O-, and S-containing functionalities, and suggests that the photoactivity of the nanoporous carbon with low functionalization for water splitting is certainly not linked to the presence of chromophoric surface groups.

Beyond surface wetting and conductivity, the carbon must have photoactive sites inside the pores at which carbon/light/water interactions can take place. Such photoreactive sites would be located at the edges of the basal planes, either associated with surface functionalities or to free edge sites linked to various configurations (carbyne-like and carbene-type) [27]. Hence upon irradiation of the carbons, excitons (holes and electrons) are photogenerated due to electronic transitions in the sp^2^ carbon clusters due to π-π* and σ-π transitions involving zig-zag, carbyne-like sites and other intermediate states [28], charge-transfer reactions in localized states involving O-, S-, and C-atoms, and/or transitions involving chromophoric groups [26,29]. The first are expected to be dominant in carbons of low functionalization such as sample NC, whereas the other might take place in highly functionalized carbon. The photogenerated electrons can be rapidly delocalized through the sp^2^ domains of the carbon anode, and transferred to electron acceptors present in the medium (i.e., oxygen, chromophores).

**Figure 2:**
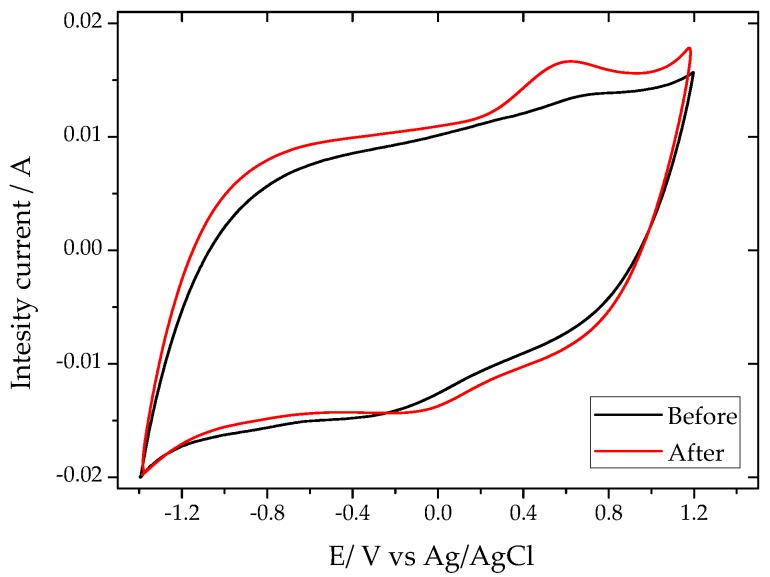
Cyclic voltammograms in 0.1 M Na_2_SO_4_ pH 2 (scan rate 20 mV/s) of the nanoporous carbon photoanode (sample NC) before and after 2 h of illumination using simulated solar light.

Given that carbon NC was the best performing photoanode (Figure 1a), in further discussion the focus of the analysis will be on this particular carbon material. To evaluate the reproducibility and stability of the electrodes under successive cycles, the photoanodes were exposed to solar irradiation for 2 h at +1 V vs. Ag/AgCl. The long-term performance of sample NC is shown in Figure 1c, and a smooth decay was observed, accounting for ca. 20% photocurrent loss after 2 h. The cyclic voltammograms (CV) of the electrode before and after the illumination are shown in Figure 2. The CV of the as-prepared electrode shows the fingerprint of a highly nanoporous carbon with a quasi-rectangular shape due to the electrical double layer capacitance of the material. Interestingly, after the electrode is exposed to solar light and/or polarization, a wide hump centered at about +0.6 V vs. Ag/AgCl appeared. The intensity of the hump is more clearly seen in the anode exposed to light, suggesting some kind of surface photosensitivity of the carbon material during the photocatalytic water splitting process, likely involving the formation of O surface groups in different environments [30], or that some specific sites of the carbon electrode might consume the photogenerated charges. Furthermore, data suggest that the occurrence of irreversible changes in the surface of the carbon during the photocatalytic process might be responsible for the drop in the long-term performance of the photoanode after 2 h of irradiation. Photocorrosion of the anodes after illumination was also observed in the functionalized carbons, and attributed to redox reactions involving the surface moieties of the carbon matrix, ultimately giving rise to the consumption of the photoactive sites and charge transfer limitations [26].

To evaluate the origin of this behavior in sample NC, the surface chemistry of the photoanodes before and after exposure to light and polarization was analyzed by temperature programmed desorption coupled to mass spectrometry, TPD-MS (Table 2 and Figure S2 in the Supplementary Materials). Complex patterns were obtained for all the electrodes (as-prepared and exposed) with unusually high amounts of CO evolved at 200 °C from the electrodes (Figure S2 in the Supplementary Materials). This indicates the existence of interferences in the quantification of the amount of CO and CO_2_ released in the electrodes, most likely arising from the carbon black and/or the binder used in the preparation of the electrodes.

Despite this, data show a clear trend with increasing amounts of O-containing groups in the carbon anodes exposed to light indicating that the carbon material is slightly oxidized (Table 2); this is more remarkable in the amount of CO_2_ desorbed, associated with the decomposition of carboxylic-like groups. Notwithstanding, to disregard the effect of the carbon additives of the electrodes, we considered a different approach for the irradiation of the carbon.

### 2.2. Surface Modification of the Nanoporous Carbon under Solar Irradiation

**Figure 3:**
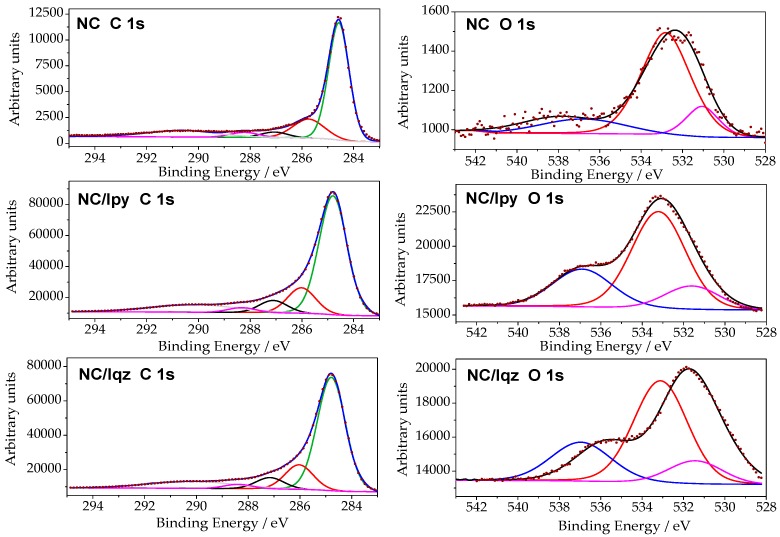
C 1*s* and O 1*s* core energy levels for the pristine nanoporous carbon (sample NC) and after irradiation using 200 < λ < 600 nm (sample NC/Iqz) and 360 < λ < 600 nm (sample NC/Ipy). Symbols correspond to experimental data, and lines to deconvolution.

To investigate the effect of the sunlight on the textural and chemical features of the carbon electrodes disregarding the interference of the binder and the carbon black conductive additive used in the fabrication of the photoanodes, an aqueous suspension of the nanoporous carbon was irradiated for 2 h. Furthermore, two different configurations were used, with filters to cut-off the irradiation below 360 nm (see experimental section), to explore the effect of the wavelength of the irradiation source on any likely changes induced in the nanoporous carbon as a result of light exposure. The characterization of the carbon material after irradiation under these conditions was carried out by XPS (Figure 3, Table 3), TPD-MS (Table 2, Figure S3 in the Supplementary Materials), and elemental analysis (Table 2).

Data from the TPD-MS study corresponding to the release of CO and CO_2_ due to the decomposition of O-groups are compiled in Table 2. As seen, a two-fold increment of the amount of CO_2_ was detected for both irradiated samples, whereas the increase in the amount of CO evolved accounted for 12%–15%. The temperature of the CO_2_ (between 200–400 °C) and CO (above 500 °C)—Figure S3 in the Supplementary Materials—desorption peaks suggests the presence of carboxylic acids and presence of phenolic and quinone like groups after the irradiation of the carbon. This fact corroborates the slight oxidation of the material upon both irradiation conditions, also detected by the elemental analysis.

The C 1*s* and O 1*s* XPS profiles and the composition of the carbon after light exposure are shown in Figure 3. Table 3 lists the results of the relative abundance of species obtained from the deconvolution of the profiles. The surface oxygen determined by XPS shows a two-fold increase in the amount of oxygen after irradiation of the carbons, with values going from 3.6 at. % for the pristine nanoporous carbon to 7.7 and 7.8 at. % for the carbon exposed to irradiation using the Iqz and Ipy configurations, respectively. This is in agreement with the data from TPD-MS and elemental analysis. This somehow contrasts with our previous work reporting the reduction of surface groups upon solar irradiation of various carbons with high surface functionalization. However, other authors have reported the oxidation of carbon materials upon exposure to irradiation [31,32,33].

Data revealed a small effect of the wavelength of the irradiation source, as both configurations showed quite similar trends, with predominance of carbonyl/quinone groups in the surface of the carbon after light exposure, accompanied by a small drop in the contribution of carboxylic groups. Although the quantification of the CO and CO_2_-evolving groups by TPD-MS showed an opposed trend (Table 2), it should be recalled that XPS accounts for the surface characterization of the material, whereas TPD-MS is a bulk technique representative of the whole material. This indicates that different redox reactions take place on the surface of the carbon, rendering a higher surface reactivity and thus functionalization.

On the other hand, the porosity of the carbon anode remained quite unmodified after light exposure, as seen in the evolution of the main textural parameters determined by gas adsorption (Table 4, Figure S4 in the Supplementary Materials).

Indeed, neither the surface area nor the distribution of pore volumes were modified upon irradiation of the carbon, regardless the wavelength of the irradiation source. A similar behavior has been observed in our previous works for several carbon materials exposed to illumination under various sources (including UV, solar, and gamma irradiations) [31,34]. Thus, the changes in the photochemical response of the anodes must be linked to the modification in the distribution of the surface groups, discarding any contribution from the porosity.

To understand this behavior, we must consider the redox reactions likely occurring upon irradiation of the carbon anodes at the carbon/water interface. As above-mentioned, in carbons of low functionalization, low to medium range excitons are formed upon light exposure due to π-π* and σ-π transitions. The anodic photocurrent detected for this carbon with the generation of oxygen confirmed the generation of reactive vacancies that react with water, producing oxygen and leaving electrons free to react with other species in the medium such as oxygen, water, or the electrode material itself. In this regard, we have confirmed the formation of O-radicals (hydroxyl and superoxide) upon irradiation of an aqueous suspension of sample NC [22]. Thus, the oxidation of the carbon electrode must be related to the photogeneration of radicals formed in the presence of water. On the other hand, it has been reported that the photogenerated electrons formed upon irradiation of nanoporous carbons with UV and solar light can reduce the surface moieties of the carbon, particularly sulfonic, quaternary nitrogen, sulfones, and carboxylic groups [26]. Reduction of the latter produces ketones and hydrogen peroxide, which can be further decomposed into hydroxyl radicals under UV light, or interact with the carbon surface to oxidize it [35]. Hence, the slight increase in CO and CO_2_-evolving groups detected by TPD-MS is due to the overall oxidation of the carbon electrode by the O-radicals. On the other hand, the surface reducing effect of the carboxylic groups is more remarkable at the carbon/water interface, where the photogenerated electrons are formed (thus explaining the relative distribution of groups detected by XPS).

The fact that we observed a similar distribution of surface groups in both lamp configurations indicates that the type of the reactions is more dependent on the nature of the carbon anode and its ability to photogenerate reactive species in solution than on the wavelength of the irradiation source.

The slight oxidation of the carbon matrix (even if subtle) is responsible for the decreased efficiency of the carbon photoanodes as photo- or electrocatalysts after long illumination periods, as high oxygen contents are detrimental for the electrical conductivity of carbon materials, and thus favor current losses.

## 3. Materials and Methods

### 3.1. Materials

A nanoporous carbon (sample referred to as NC) obtained from CO_2_ activation of bituminous coal was selected for the preparation of the photoanodes. The choice of the material was based on its reported photochemical activity towards the oxidation of pollutants in solution [20,23]. The pristine carbon was chemically modified by wet oxidation using saturated (NH_4_)_2_S_2_O_8_ in 4 N H_2_SO_4_ at room temperature (ca. 1 g of carbon per 10 mL solution) and stirred overnight (sample NCox). For the preparation of the photoanodes a slurry of the nanoporous carbon, polyvinylidene difluoride as binder, and a carbon black conductive additive (ratio 85:10:5) in *N*-methyl-2-pyrrolidone was coated on a 1 cm^2^ Ti foil collector. The electrodes were dried in air at 120 °C before usage. For comparison purposes, the photocatalytic performance of the photoanode prepared from sample NC was compared to that of other nanoporous carbons from a previous study (samples CONS; CONS-I; and a wood-based commercial carbon, sample BAX) [26].

### 3.2. Photocatalytic Measurements

A standard three-compartment cell with an optically flat quartz window on the side and consisting of the nanoporous carbon photoanode as working electrode, a graphite rod as counter electrode, and a saturated Ag/AgCl as reference electrode were used. A solar simulator (irradiation density of 75 W/cm^2^) was used as irradiation source; a pyrex filter (cut-off below 360 nm) was employed to isolate the contribution of visible light in the solar simulator. The photoanodes were immersed in 20 mL of an aqueous electrolyte (0.1 M Na_2_SO_4_, pH adjusted to 2) and purged with N_2_ before the illumination. The transient photocurrents of the carbon photoanode under on/off illumination were recorded at constant potentials between 0 and +1.5 V vs. Ag/AgCl, in a potentiostat (Biologic). Dark current equilibrium at the bias potential was allowed before the irradiation. The O_2_ concentration in the electrochemical cell was measured using an oxygen sensor immersed in the electrolyte. The cyclic voltammetries, at a potential rate of 20 mV/s, of the carbon photoanodes before and after light exposure were also recorded.

### 3.3. Irradiation Conditions for Surface Modification

To evaluate the surface modification of the carbon photoanode by the effect of the solar irradiation, water suspensions of powders of the carbon material (ca. 1 g/L loading) were exposed to irradiation for 2 h under continuous stirring. The photoreactor consisted of a high pressure mercury lamp (Helios Italquartz, 125 W) surrounded by a cylindrical, double-walled jacket cooled by flowing water (to prevent overheating). Two different irradiation conditions were studied to evaluate the wavelength dependence of the surface modification: the photoreactor with a quartz jacket (irradiation Iqz corresponding to 200 < λ < 600 nm, or a pyrex jacket as a cut-off filter (irradiation Ipy, 360 < λ < 600 nm). The incident photon flux in the photoreactor was measured by chemical actinometry [36] in both configuration conditions, with values of 9.1 × 10^−6^ and 1.35 × 10^−5^ Einstein/s for Iqz and Ipy, respectively. The nomenclature of the irradiated samples will be NC/Z where Z stands for the code assigned to the irradiation conditions (Iqz, Ipy).

### 3.4. Textural Characterization

Before the characterization, the carbons were degassed under vacuum at 393 K for 17 h. The high-resolution adsorption isotherms of CO_2_ and N_2_ at 273 and 77 K, respectively, were recorded in volumetric analyzers (Micromeritics). The N_2_ data was used to calculate the specific surface area (S_BET_) and total pore volume (V_total_). The narrow microporosity was evaluated from the CO_2_ data using the Dubinin-Radushkevich formulism. Ultrahigh purity gases were provided by Air Products.

### 3.5. Chemical Characterization

Samples were chemically characterized by elemental analysis. The determination of carbon, hydrogen, and nitrogen was carried out by a LECO CHNS-932 (ASTM D-5373), while oxygen was directly measured in a LECO VTF-900 CHNS-932 microanalayzer. The samples were dried under vacuum at 393 K for 17 h before the analysis. Temperature programmed desorption (TPD) was measured in a chemisorption analyzer (Autochem 292) connected to a mass spectrometer (TPD-MS) for gas analysis, obtaining the gas evolution profiles as a function of temperature. About 10 mg of carbon sample were heated up to 1073 K (10 K/min) under a constant argon flow (50 mL/min). The composition of the evolved gases (including the quantification of the amounts of CO and CO_2_) was followed by the mass spectrometer. XPS for the analysis of the C 1*s* and O 1*s* core level signals were collected in a SPECS spectrometer by using Al Kα (1486.74 eV) radiation with a power of 100 W in the constant analyzer energy mode. Spectra of dried powdered samples were recorded with constant pass energy values of 50 eV, using a 720 μm diameter analysis area. During the processing of the XPS spectra, energy values were referenced to the C 1*s* peak of adventitious carbon located at 284.6 eV. The PHI ACCESS ESCA-V6.F software package was used for acquisition and data analysis. A Shirley-type background was subtracted from the signals. Recorded spectra were always fitted using Gauss–Lorentz curves, in order to determine the binding energy of the different element core levels more accurately. The error in BE was estimated to be ca. 0.1 eV.

## 4. Conclusions

We have explored the origin of the photocorrosion phenomenon observed in nanoporous carbon photoanodes showing low surface functionalization upon long-term exposure to solar light, by studying the changes in the specific textural, chemical, and structural features of the carbon material after irradiation under different conditions (UV and visible light). The nanoporous carbon photoanode exhibited good performance towards the oxygen evolution reaction under simulated solar light, with an onset of the photocurrent at a low overpotential (ca. +0.8 V vs. Ag/AgCl). The loss in efficiency after 2 h of irradiation suggested the occurrence of photo- and/or electro-corrosion of the anode, as already reported for highly functionalized carbon anodes. As opposed to the latter, where the drop-in performance was attributed to the reduction of some groups in the carbon surface, the characterization of the carbon photoanode revealed that the photocorrosion of carbon anodes of low functionalization proceeded through a different pathway. The carbon matrix gets slightly oxidized, with the formation of carboxylic and carbonyl-like moieties, whereas the porosity remained unchanged. The oxidation of the carbon matrix occurred due to the oxygen reactive species formed upon the decomposition of water during the irradiation of the photoanode. Similar surface modifications of the carbon were obtained in both lamp configurations, indicating that the photoinduced surface reactions depend on the nature of the carbon anode and its ability to photogenerate reactive species in solution, rather than on the wavelength of the irradiation source. The surface changes upon irradiation of the carbon photoanode are responsible for its decreased efficiency throughout long illumination periods, due to the effect of the oxidation of the carbon matrix on the conductivity.

## Figures and Tables

**Table 1:** Textural characteristics of the investigated carbon electrodes obtained from N_2_ adsorption isotherms at 77 K.

Samples	S_BET_ (m^2^/g)	V_total_ (cm^3^/g) ^A^	V_micropores_ (cm^3^/g) ^B^	V_mesopores_ (cm^3^/g) ^B^
NC	1027	0.524	0.320	0.090
NCox	989	0.500	0.310	0.070
BAX	2010	1.463	0.472	0.991
CONS	38	0.022	0.001	0.021
CONS-I	727	0.363	0.260	0.103

^A^ Evaluated at p/p_0_ 0.99; ^B^ Evaluated using the 2D-NLDFT method.

**Table 2:** Quantification of the amount of CO and CO_2_ evolved in the TPD-MS profiles and oxygen amount obtained by elemental analysis of the photoanodes exposed to illumination and/or polarization and the nanoporous carbon powders irradiated in water.

Samples	CO_2_ Evolved (mmol/g)	CO Evolved (mmol/g)	Oxygen (wt. %) ^A^
NC photoanode (as prepared)	0.320	1.139	1.9
NC photoanode irradiated (2 h)	0.502	1.251	--
NC photoanode irradiated (2 h) and polarized (1 V vs. Ag/AgCl)	0.555	1.603	--
NC (pristine carbon)	0.164	0.557	1.9
NC/Ipy ( irradiation 360 < λ < 600 nm)	0.292	0.630	6.9
NC/Iqz (irradiation 200 < λ < 600 nm)	0.282	0.652	5.8

^A^ evaluated from elemental analysis.

**Table 3:** Relative distribution of surface groups (%) obtained by deconvolution of XPS data corresponding to C 1*s* and O 1*s* core energy level. Quantification (at. %) of the overall carbon and oxygen contents is also included for clarity.

	NC	NC/Iqz	NC/Ipy
–C–C in graphitic carbon (284.3–284.9 eV)	64.2	64.6	64.2
–C–(O, S, H) in phenolic, alcoholic (285.2–286.0 eV)	15.8	14.0	13.7
–C=O in carbonyl or quinone (286.56–287.1 eV)	3.9	6.4	6.7
–O–C=O in carboxyl or ester (287.6–288.22 eV)	3.8	2.4	2.5
–carbonate, occluded CO, ð-electrons in aromatic ring (289.3–289.5 eV)	12.3	12.6	12.9
Total C 1*s* (at. %)	96.4	92.3	92.2
–C=O in carbonyl, quinone (530.6–531.9eV)	13.8	24.1	23.9
–C–O in phenol/epoxy, ether, thioethers (532.3–533.9 eV)	70.3	64.9	49.5
–O– in carboxyl, water or chemisorbed oxygen species (534.7–535.0 eV)	15.9	11.0	26.6
Total O 1*s* (at. %)	3.6	7.7	7.8

**Table 4:** Textural characteristics of the studied carbons before and after irradiation, obtained from N_2_ and CO_2_ adsorption isotherms at 77 and 273 K, respectively.

Samples	S_BET_ (m^2^/g)	V_total_ (cm^3^/g) ^A^	W_0,_ N_2_ (cm^3^/g) ^B^	W_0_, CO_2_ (cm^3^/g) ^B^
NC (pristine carbon)	1027	0.524	0.386	0.197
NC/Iqz ( irradiation 200 < λ < 600 nm)	1018	0.537	0.488	0.168
NC/Ipy (irradiation 360 < λ < 600 nm)	1033	0.579	0.580	0.170

^A^ Evaluated at p/p_0_ 0.99; ^B^ Evaluated using the DR method.

## Supplementary Materials

Supplementary materials can be accessed at: http://www.mdpi.com/1420-3049/21/11/1611/s1.
