# Supplementary material for: Surface Modification of a Nanoporous Carbon Photoanode upon Irradiation
Source: Molecules. 2016 Nov 23;21(11):1611. doi: 10.3390/molecules21111611 (PMC6273716; doi:10.3390/molecules21111611)
Supplement: Supplementary file 1 [file molecules-21-01611-s001.pdf]

Molecules **2016**, *21*, 1611; doi:10.3390/molecules21121611

Supplementary Materials: Surface Modification of a Nanoporous Carbon Photoanode upon Irradiation

Alicia Gomis-Berenguer, Inmaculada Velo-Gala, Enrique Rodríguez-Castellón and Conchi O. Ania

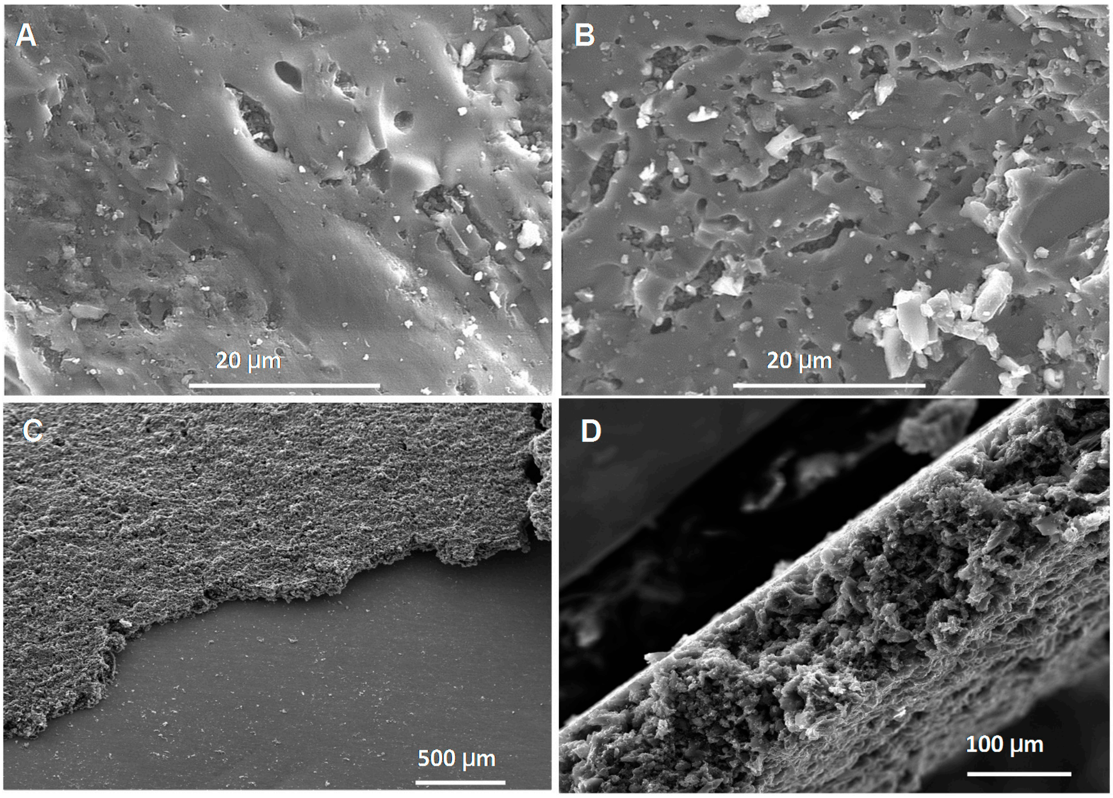

Figure S1. SEM images of the investigated carbon materials (**A**) NC and (**B**) NCox, and examples of the photoanodes (**C**, **D**).

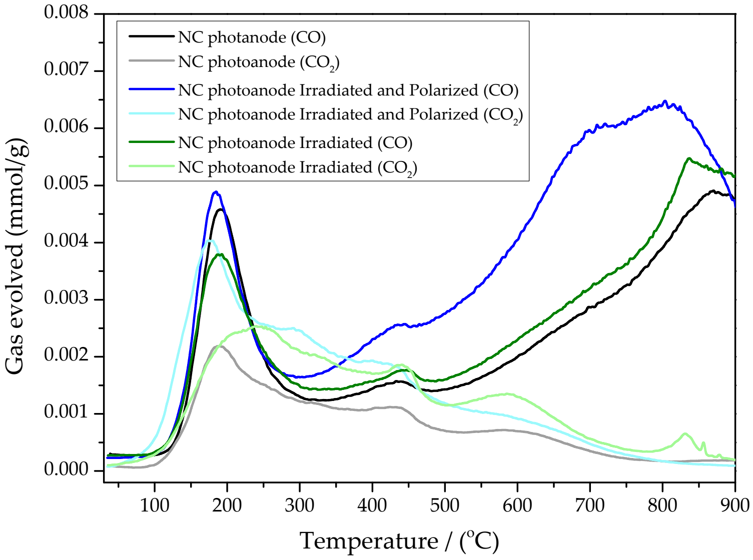

Figure S2. CO and CO_2 TPD-MS profiles for the as prepared carbon photoanode, and after irradiation and polarization at 1 V vs Ag/AgCl.

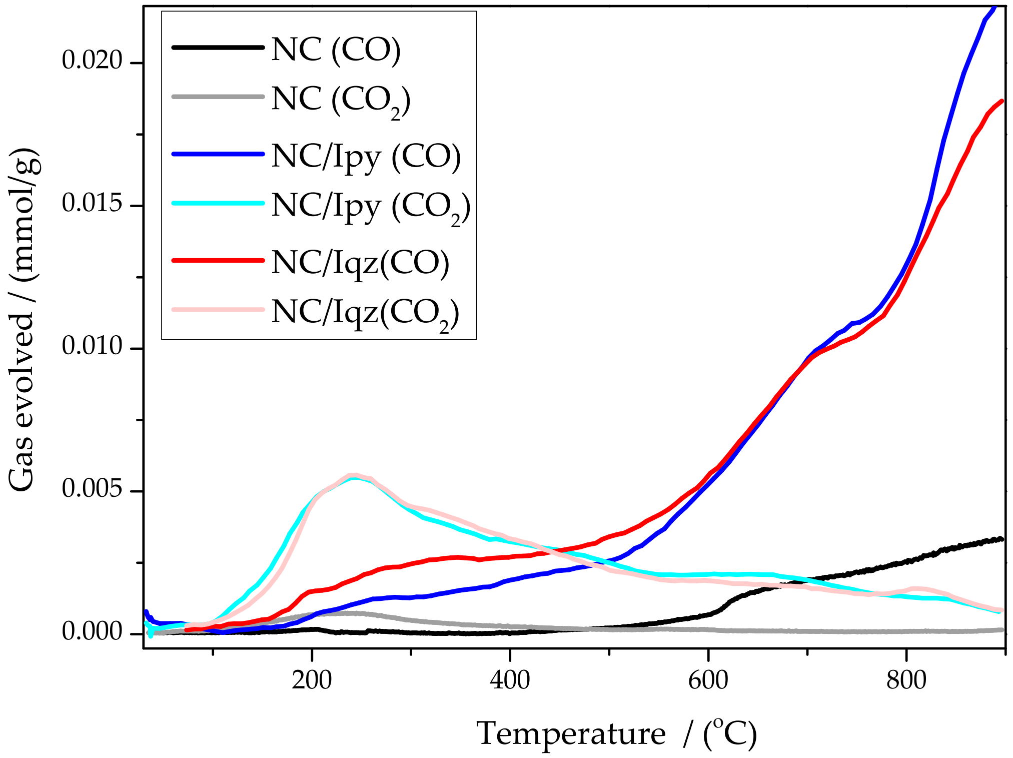

Figure S3. CO and CO_2 TPD-MS profiles for the nanoporous carbon before and after light exposure with different irradiation conditions.

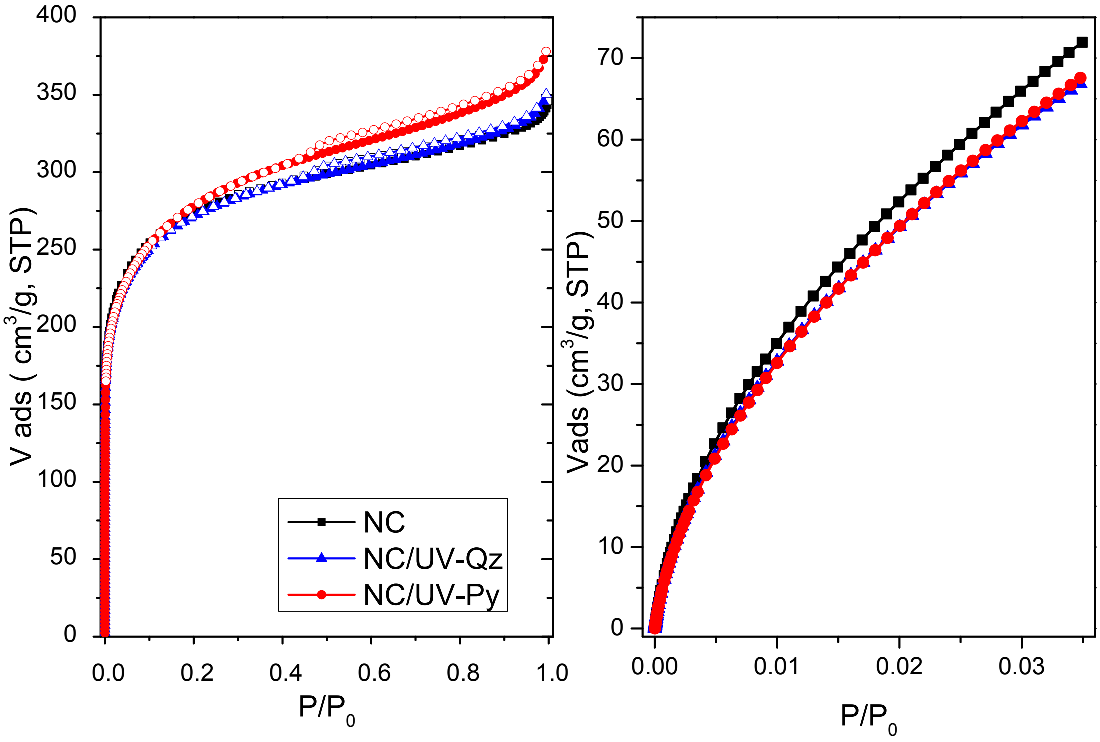

Figure S4. High resolution adsorption/desorption isotherms of the studied nanoporous carbons: (**left**) nitrogen at 77K, and (**right**) carbon dioxide at 273K.
